# Remote Monitoring Activity Trajectory-Associated Weight Loss and Functional Ability in Obese Older Adults

**DOI:** 10.1093/geroni/igab046.199

**Published:** 2021-12-17

**Authors:** John Batsis, Curtis Petersen, Anna Kahkoska, Karen Fortuna

**Affiliations:** 1 University of North Carolina at Chapel Hill, Chapel Hill, North Carolina, United States; 2 Dartmouth College, Lebanon, New Hampshire, United States; 3 University of North Carolina at Chapel Hill, University of North Carolina at Chapel Hill/Chapel Hill, North Carolina, United States

## Abstract

Functional decline in older adults can often be mitigated by physical activity. As older adults increasing adopt wearable technology, an understanding of how remotely monitored activity is associated with clinical outcomes is needed. Data was analyzed from two cohorts of older adults with obesity (□65 years, BMI (□30kg/m2)) who completed weekly dietary and exercise-based weight loss interventions (n=93). Follow-up time varied between cohorts (n=37: 12-weeks; n=56: 16 weeks). All participants were provided a Fitbit to monitor physical activity. Baseline and follow up weight, 6-minute walk distance, grip strength, and Late Life Function and Disability Instrument (LLFDI) were collected. We used k-means clustering for longitudinal data to identify physical activity trajectories from Fitbit steps at the daily level. Linear regression models tested for differences in each outcome between trajectories, adjusting for age and sex. Baseline characteristics did not vary across cohorts: mean age 72.7±4.5 years, 76.5% were female, and mean BMI was 36.4±5.1 kg/m2. Two physical activity trajectories were identified, termed high and low activity based on differences in mean daily steps (7,476±4,117 vs. 2,960±2,453, p <0.001). Participants in the high activity group experienced a 2.4% reduction in weight (p <0.001) and a 4.74% increase in LLFDI score (p=0.007) relative to the low activity cluster. Other outcomes were not significantly different between trajectories. These results demonstrate the potential for remote monitoring data to elucidate longitudinal trends in weight and functional ability. As such, older adults’ use of wearable technology may facilitate improvements in weight and functional ability in the community.

